# Systematic risk analysis of radiation pneumonitis in breast cancer: role of cotreatment with chemo-, endocrine, and targeted therapy

**DOI:** 10.1007/s00066-022-02032-y

**Published:** 2022-12-14

**Authors:** Julian Mangesius, Danijela Minasch, Katharina Fink, Meinhard Nevinny-Stickel, Peter Lukas, Ute Ganswindt, Thomas Seppi

**Affiliations:** Department of Radiation Oncology, Medical University of Innsbruck, 6020 Innsbruck, Anichstr. 35, Austria

**Keywords:** Radiation pneumonitis, Breast cancer, Radiation therapy, Lung toxicity, Goserelin, Tamoxifen, Organs at risk, Normal tissue complications

## Abstract

**Purpose:**

A major complication of sequential and concomitant chemoradiation in breast cancer treatment is interstitial pneumonitis induced by radiation therapy (RT), systemic therapy, or a combination of both. Dose and volume of co-irradiated lung tissue directly correlate with the risk of radiation pneumonitis. Especially in case of combined treatment, it is often unclear which of the used therapeutic agents promote pneumonitis.

**Methods:**

This was a prospective monocentric study including 396 breast cancer patients. A systematic analysis of single and combined therapeutic measures was performed in order to identify treatment-related factors enhancing the risk of pneumonitis post RT.

**Results:**

Overall incidence of pneumonitis of any grade was 38%; 28% were asymptomatic (grade 1) and 10% were symptomatic (> grade 1). Pneumonitis > grade 2 did not occur. Beside age, smoking status, and mean lung dose, the combined treatment with goserelin and tamoxifen significantly enhanced the risk of pneumonitis in a supra-additive pattern (odds ratio [OR] 4.38), whereas each agent alone or combined with other drugs only nonsignificantly contributed to a higher pneumonitis incidence post RT (OR 1.52 and OR 1.16, respectively). None of the other systemic treatments, including taxanes, increased radiation pneumonitis risk in sequential chemoradiation.

**Conclusion:**

Common treatment schedules in sequential chemoradiation following breast-conserving surgery only moderately increase lung toxicity, mainly as an asymptomatic complication, or to a minor extent, as transient pneumonitis ≤ grade 2. However, combined treatment with tamoxifen and the LHRH analog goserelin significantly increased the risk of pneumonitis in breast cancer patients after chemoradiation. Thus, closer surveillance of involved patients is advisable.

## Introduction

Breast cancer is one of the most common malignancies, with an 11.7% global incidence of all diagnosed malignancies [1]. Breast-conserving surgery followed by radiotherapy is the most prevalent therapy. Especially asymptomatic radiation pneumonitis (RP) but also symptomatic RP are known lung complications triggered by breast cancer radiotherapy.

Overall mortality from RP and recall radiation pneumonitis (RRP) is commonly reported to be less than 2%. However, higher grade (≥ 2) pneumonitis is associated with significant impairment of quality of life, prolonged corticosteroid treatment, and it may progress to irreversible lung injury such as fibrosis and organizing pneumonia [2]. The onset of clinical manifestation can occur within a few weeks or months (RP), and up to several years following RT (RRP) [3].

Early studies reporting on orthovoltage treatment showed an occurrence of pneumonitis of up to 35% [4], with declining incidences over the following decades [5, 6]. Meanwhile, a strong relationship between RP development and the applied breast cancer treatment has become evident. Differences in RP incidences have been reported for standard 3D tangential field irradiation, VMAT, and IMRT [7, 8]. It is accepted knowledge that a cumulative dose deposition of > 20 Gy to 20% of the total lung volume (V_20Gy_) significantly correlates with an elevated RP risk [9]. The surrogate parameter central lung distance (CLD), widely used in the past to estimate the co-irradiated lung volume, has largely become obsolete with emerging computed planning techniques including precise dose–volume histogram calculations to minimize normal tissue complications. Meanwhile, there is evidence that CLD is of limited value in RP risk prediction and, if at all, is applicable to breast and chest wall irradiation only [6, 10].

Recently, we were able to demonstrate that calculation of mean lung dose (MLD) is the most reliable predictor of RP risk after breast cancer RT. The incidence of RP rose significantly at MLD values greater than 10 Gy. Of all investigated factors, only MLD showed a direct and reliable correlation to the rate of asymptomatic and symptomatic pneumonitis in a cohort of 100 consecutively treated patients [11].

Independently of radiotherapy, common chemotherapeutics and novel anti-cancer agents have also been investigated for their potential to induce toxicities in organs at risk [12–14], including the lung. Drug-related pneumonitis is reported to be induced by chemotherapy (taxanes, gemcitabine, bleomycin, etc.), by targeted agents (anti-PD-1/PD-L1 mAbs, EGFR inhibitors, mTOR inhibitors, etc.), and by combined drug regimens [15–17]. Among chemotherapeutics, especially taxanes are controversially discussed as independent inducers of lung toxicities [18–21], either administered as dose-dense monotherapy or in combination with other drugs and radiotherapy. In addition, molecular mechanisms of drug-related pneumonitis are often unknown, especially concerning novel substances and antibodies—even more so if combined with radiotherapy.

Early and late responses of lung tissue following adjuvant hormonal therapy are mainly reported for patients receiving tamoxifen [22]. Finally, anticancer drugs have been associated with rarely observed radiation recall pneumonitis including gemcitabine, paclitaxel, anthracyclines, trastuzumab, and everolimus [23].

However, the available data on lung complications derived from radiation therapy combined with systemic treatments are largely based on case reports, retrospective studies with a limited number of patients, or on studies not yet including dose–volume analyses of co-irradiated lung tissue. Consequently, the distinct contribution of radiotherapy and of systemic treatment on RP incidence could not be differentiated reliably in combined treatment regimens so far.

In order to gain maximum benefit from combined treatment schedules, it is essential to accurately estimate individual patients’ risk for developing radiation pneumonitis prior to treatment. For this purpose, a quantitative contribution assessment of pre-existing patient risk factors, of delivered MLD, and of varying adjuvant therapies is a prerequisite. Thus, this prospective study aims to systematically evaluate and weigh up inherent as well as treatment-associated parameters determining risk and incidence of lung toxicities in breast cancer treatment.

## Patients and methods

### Study design and patient characteristics

The presented study including 400 breast cancer patients was a prospective monocentric and observational explorative investigation to assess the contribution of radiotherapy and systemic therapy to the incidence of radiation pneumonitis. The institutional ethics committee has approved the study, and all enrolled patients provided written informed consent. Patient recruitment occurred between 01/2013 and 07/2018 (with a recruitment rate of approximately 25% of all patients receiving adjuvant breast cancer irradiation), with a follow-up time of 24 months.

Eligible patients were required to be aged 18 years or older and to exhibit histologically evident adenocarcinoma or carcinoma in situ excised by breast-conserving surgery. Enrolment was restricted to patients receiving their entire prescribed total dose of unilateral total breast radiotherapy with or without nodal co-irradiation. Both radiation therapy alone as well as neoadjuvant and adjuvant systemic therapies of any kind were admissible. Patients with a history of prior radiation therapies were excluded. Four initially participating patients retracted their informed consent before the first study follow-up visit. Thus, 396 of the 400 recruited patients were finally included in data analyses.

### Radiotherapy

Radiotherapy was initiated within 6 weeks after surgery or after adjuvant systemic therapy. If indicated, treatment of the unilateral breast and chest wall (BCW; *N* = 285; 72.0%) was extended to the locoregional nodal areas, paraclavicular (BCW + PCN; *N* = 34; 8.6%) and/or internal mammary (BCW + PCN + IMN, *N* = 77; 19.4%). The 3D treatment planning was performed using Pinnacle V.14 (Philips Healthcare, Amsterdam, Netherlands). Therapy was delivered by Elekta Versa Linac (Synergy, Precise and Versa HD linear accelerators, Elekta AB, Stockholm, Sweden). Individual organ at risk (OAR) dose constraints were assessed using cumulative dose–volume histograms (DVH). Total lung dose (TLD) load in the case of PCN or PCN + IMN inclusion was limited to V_20Gy_ of ≤ 30% and a V_30Gy_ of ≤ 20%, whereas in the case of BCW irradiation only, it was limited to a V_20Gy_ ≤ 25%. Patients with carcinoma in situ were given a total dose of 50 Gy (6 MeV, 2 Gy/day, in five fractions per week, *N* = 70; 17.7%), whereas the total dose for an invasive carcinoma of the breast was set at 56 Gy (*N* = 326; 82.3%) [24–26], in accordance with local guidelines and institutional practice. If indicated, a sequential boost to the tumor bed to a target volume dose of 60 Gy was applied (*N* = 85; 21.5%). Locoregional nodal areas were treated with a total dose of 50 Gy.

### Study procedures and outcome measures

Three planned follow-up visits were performed: at therapy end and at week 12 and week 25 after radiotherapy. Clinical evaluation included monitoring of adverse events, medication changes, newly diagnosed respiratory diseases, and clinical symptoms like dyspnea, dry cough, and thoracic pain. Thoracic CT scans and blood sample analyses were performed 12 and 25 weeks after treatment to assess the primary endpoint of asymptomatic and symptomatic radiation pneumonitis. In case of clinical symptoms indicative of radiation pneumonitis, an immediate unscheduled visit was performed. Evidenced symptomatic pneumonitis was treated by a standardized course of steroid administration. These patients were continuously monitored until respiratory impairment was alleviated or overcome.

Grading of lung complications was performed following the Common Terminology Criteria for Adverse Events (CTCAE). Observed CT alterations were identified as radiation pneumonitis if ground-glass opacities and/or airspace consolidation were found within the co-irradiated lung volume. Radiological findings without clinical symptoms were classified as asymptomatic RP (CTCAE grade 1). Radiation pneumonitis was classified as symptomatic if CT alterations were accompanied by non-productive cough, newly appearing or deterioration of pre-existing dyspnea, thoracic pain, fever, or malaise (CTCAE grade ≥ 2).

Systematic monitoring of potential risk factors for developing radiation pneumonitis was performed, including age, KPS, BMI, MLD, CLD, systemic therapies including endocrine treatment, smoking status, pre-existing lung conditions, allergies, nodal area cotreatment, boost irradiation, and affected breast side.

### Systemic therapy

In all, 355 patients had completed various single or combined chemotherapeutic, endocrine, and/or antibody regimens as neoadjuvant or adjuvant treatment prior to radiotherapy (Table 1). Chemotherapeutics administered to 136 patients comprised anthracyclines (epirubicin, doxorubicin; *N* = 115), carboplatin (*N* = 51), taxanes (docetaxel, paclitaxel; *N* = 122), cyclophosphamide (*N* = 50), 5‑fluorouracil (5-FU, *N* = 40), or a combination of these. Antibody regimens were performed in 61 patients using anti-HER2 trastuzumab or pertuzumab, or a combination of both. Antihormonal therapy was performed in 290 patients with the estrogen receptor blocker tamoxifen (*N* = 83), aromatase inhibitors (anastrozole or letrozole; *N* = 188), and GnRH analogs (leuprorelin or goserelin; *N* = 60), either alone or in combination. Indication and dosage setup of all administered therapeutics was performed according to S3 and the ABCSG guidelines.

**Table 1:** Patient (*N* = 396) and treatment characteristics—incidences of radiation pneumonitis

	Symptomatic RP				Any-grade RP (symptomatic and asymptomatic)			
	No RP (*N* = 356)	RP (*N* = 40)	% RP	*p-*value	No RP (*N* = 245)	RP (*N* = 151)	% RP	*p*-value
*Age*	–	–	–	0.215	–	–	–	0.009
Median	56 (24–83)	60.5 (29–79)	–	–	56 (24–83)	57 (29–81)	–	–
*MLD [Gy]*								
Mean ± SD	5.1 ± 2.8	6.6 ± 3.8	–	0.009	4.6 ± 2.4	6.3 ± 3.3	–	< 0.0001
Median (range)	4.2 (1.1–15.1)	5.6 (2.1–19.4)	–	–	3.9 (1.1–15.1)	5.2 (1.1–19.4)	–	–
*CLD*								
Mean ± SD	21.2 ± 4.8	22.9 ± 7.0	–	0.134	20.4 ± 4.7	22.9 ± 5.4	–	< 0.001
Median (range)	21 (10–48)	21.5 (10–45)	–	–	20 (10–48)	23 (10–45)	–	–
*Time to radiologic recovery [months]*								
Mean ± SD	–	10.7 ± 6.7	–	–	–	6.6 ± 5.3	–	–
Median (range)	–	9.5 (1–24)	–	–	–	3 (1–24)	–	–
*RT target regions*	–	–	–	0.002	–	–	–	< 0.001
BCW	264	21	7.4	–	195	90	31.6	–
BCW + PCN	30	4	11.8	–	16	18	52.9	–
BCW + PCN + IMN	62	15	19.5	–	34	43	55.8	–
*Boost RT*	–	–	–	0.567	–	–	–	0.515
Yes	75	10	11.8	–	50	35	41.2	–
No	281	30	9.6	–	195	116	37.3	–
*Side*	–	–	–	0.779	–	–	–	0.670
Left	177	21	10.6	–	124	74	37.4	–
Right	178	19	9.6	–	120	77	39.1	–
Bilateral	1	0	0.0	–	1	0	0.0	–
*Combined therapy*	–	–	–	0.469	–	–	–	0.178
RT alone	35	6	14.6	–	27	14	34.1	–
RT + CTX	34	1	2.9	–	25	10	28.6	–
RT + CTX + AB	25	3	10.7	–	14	14	50.0	–
RT + CTX + ET	44	2	4.3	–	31	15	32.6	–
RT + HT	189	24	11.3	–	132	81	38.0	–
RT + AB	2	0	0.0	–	1	1	50.0	–
RT + ET + AB	3	1	25.0	–	2	2	50.0	–
RT + CTX + ET + AB	24	3	11.1	–	13	14	51.9	–
*MLD group*	–	–	–	0.001	–	–	–	< 0.001
< 5 Gy	234	19	7.5	–	180	73	28.9	–
5–7.5 Gy	52	5	8.8	–	29	28	49.1	–
7.5–10 Gy	45	8	15.1	–	26	27	50.9	–
> 10 Gy	25	8	24.2	–	10	23	69.7	–
*KPS reduction*	41 (12.5%)	15 (37.5%)	–	< 0.001	28 (12.8%)	28 (18.8%)	–	0.116
*Smoking status*								
Nonsmokers	210	23	9.9	–	139	94	40.3	–
Former smokers (vs nonsmokers)	46	5	9.8	0.988	35	16	31.4	0.028
Active smokers (vs. nonsmokers)	72	7	8.9	0.793	58	21	26.6	0.012
Active or former smokers (vs. nonsmokers)	118	12	9.2	0.843	93	37	28.5	0.024
*History of pneumonia*	–	–	–	0.313	–	–	–	0.843
Yes	21	4	16.0	–	15	10	40.0	–
No	335	36	9.7	–	230	141	38.0	–
*Chronic lung disease*	–	–	–	0.517	–	–	–	0.827
Yes	28	2	6.7	–	18	12	40.0	–
No	328	38	10.4	–	227	139	38.0	–
*Allergies*	–	–	–	0.814	–	–	–	0.339
Yes	122	13	9.6	–	79	56	41.5	–
No	233	27	10.4	–	165	95	36.5	–

*RP* radiation pneumonitis, *MLD* mean lung dose, *CLD* center lung distance, *BCW* breast and chest wall, *PCN* paraclavicular nodes, *IMN* internal mammary nodes, *CTX* chemotherapy + *ET* endocrine therapy + *AB* antibody therapy

Combined chemotherapy schedules included: 1) epirubicin 90 mg/m^2^, cyclophosphamide 600 mg/m^2^ (EC), docetaxel 100 mg/m^2^ on day 1, every 21 days for four cycles (*N* = 31; EC without docetaxel *N* = 4); 2) 5-FU 500 mg/m^2^, epirubicin 100 mg/m^2^, and cyclophosphamide 500 mg/m^2^ (FEC) on day 1 every 21 days for three cycles, followed by docetaxel 100 mg/m^2^ on day 1 every 21 days for three cycles (*N* = 36; FEC without docetaxel for six cycles *N* = 4); 3) liposomal doxorubicin 50 mg/m^2^ and docetaxel 60 mg/m^2^ on day 1 every 21 days and trastuzumab/pertuzumab weekly for six cycles (*N* = 27); 5) carboplatin AUC 6 and docetaxel 75 mg/m^2^, day 1 every 21 days for six cycles and trastuzumab adjuvant over 1 year (*N* = 12).

For statistical analysis, SPSS Statistics (version 26, IBM Corp., Armonk, NY, USA) was used. Descriptive statistics were performed for all variables of interest. Associations between dichotomous risk variables and RP were analyzed with Pearson’s chi-square test. Continuous risk variables were analyzed using Spearman’s rank correlation. Comparisons of MLD between RP and non-RP patients were performed using independent samples *t*-test. Normality of data was assessed with Kolmogorov–Smirnov test. Univariate analysis of systemic therapies as predictors for RP risk was performed using binary logistic regression. Results were considered significant if *p* < 0.05.

## Results

Overall incidence of radiation-induced pneumonitis of any grade was 38% (151/396), 28% (111/396) were asymptomatic with radiologic findings only (grade 1) and 10% (40/396) were symptomatic (> grade 1). RP exceeding grade 2 according to CTCAE v5.0 was not observed in our cohort. Consequently, none of the patients needed hospitalization or died of radiation pneumonitis. Mean time to radiologic recovery was 6.6 months (SD = 5.3).

Incidences of radiation pneumonitis and patient and treatment characteristics are displayed in Table 1. Analysis of patient-inherent risk factors showed no significant correlation between any-grade RP and the Karnofsky performance status (*p* = 0.74) or history of chronic lung disease (*p* = 0.83), pneumonia (*p* = 0.84), or allergies (*p* = 0.34). RP incidence was significantly elevated for older patients (*p* < 0.01) and nonsmokers (*p* = 0.02). No correlation between any inherent risk factor and symptomatic (grade > 1) RP was detected (Table 1).

Regarding RT-related parameters, a significant correlation between MLD and incidences of any-grade RP (rs = 0.265, *p* < 0.0001) and symptomatic RP (rs = 0.12, *p* = 0.009) was detected. The corresponding surrogate parameter CLD significantly correlated with any-grade RP (*p* < 0.001) but not with symptomatic RP (*p* = 0.13). Extension of RT to the locoregional nodal areas (paraclavicular and IMN) significantly correlated with increased risks of symptomatic (*p* = 0.002) and any-grade RP (*p* < 0.001). The treated body side (*p* = 0.67) as well as boost irradiation (*p* = 0.52) had no effect on RP risk.

**Fig. 1:**
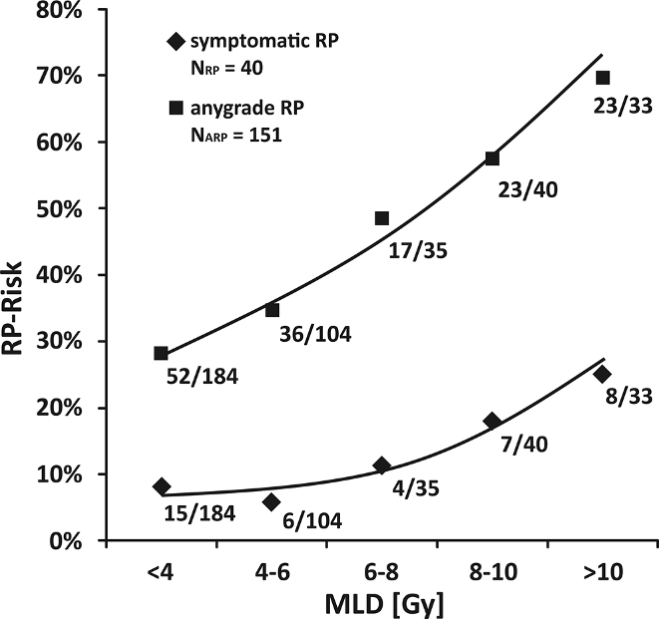
Risk of lung complications of breast cancer patients as a function of increasing mean lung dose (*MLD*). Irrespective of administered combined systemic schedules, the risk of developing clinically apparent radiation pneumonitis (RP; symptomatic grade 2—*diamond pattern*) and the risk of developing any-grade RP (asymptomatic grade 1 and symptomatic grade 2 together, *square pattern*) was assessed based on observed incidences in the cohort of 396 patients

The risk of developing RP following radiotherapy of breast cancer is a direct function of increasing mean lung dose. In fact, patients’ risk of developing symptomatic RP ranges from 8% at MLD < 4 Gy to 24% at MLD > 10 Gy. An analogous association is observed for incidences of any-grade RP (28% at MLD < 4 Gy to 70% at MLD > 10 Gy; Fig. 1). If compared to breast-and chest wall irradiation (BCW), the incidence of symptomatic pneumonitis increased by a factor of 1.6 after inclusion of the supraclavicular nodal region (BCW + PCN), and by a factor of 2.6 after inclusion of both supraclavicular and parasternal nodes (BCW + PCN + IMN). These increases in RP incidence correspond to mean MLD increases by a factor of 1.9 following treatment inclusion of PCN, and by a factor of 2.6 including PCN and IMN. By considering the observed relationship, an exponential increase in RP rates along linear increases of MLD is confirmed, as previously reported [11]. Second, the exponentially increasing rates of RP incidences within the three treatment sub-cohorts (BCW/BCW + PCN/BCW + PCN + IMN) directly correlate with correspondingly increasing mean MLD. Almost all cases of MLD > 7.5 Gy were observed in patients treated by inclusion of PCN or PCN + IMN (84 of 86 patients, 98%).

**Fig. 2:**
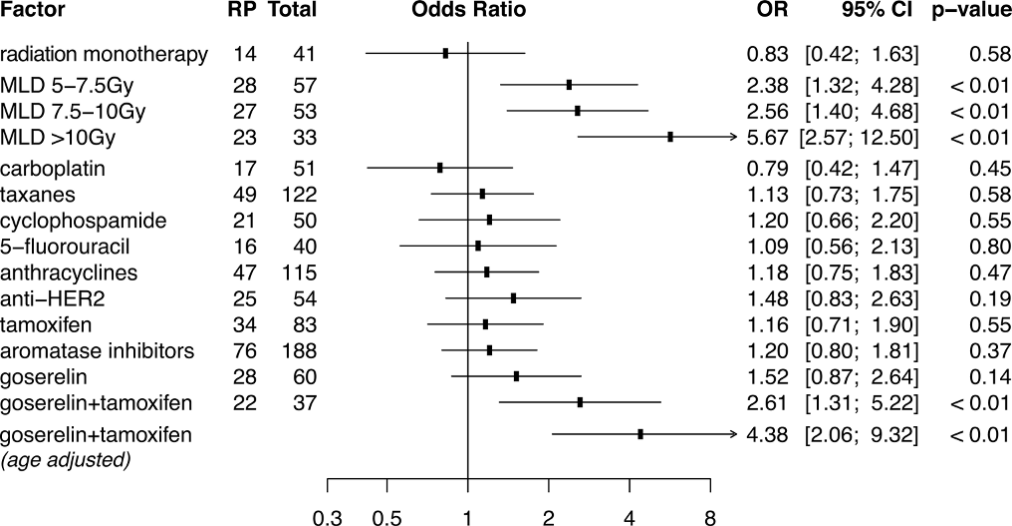
Risk of any-grade radiation pneumonitis depending on administered adjuvant therapeutics and co-irradiation of the lung. The odds of developing any-grade RP of each treatment subgroup in reference to all other treatments are displayed. The relative risk for RP development depending on MLD was referenced to patients exposed to less than 5 Gy MLD each

Combination of RT with chemotherapy and antibodies, or combinations thereof, did not correlate with either symptomatic or any-grade RP incidence (Table 1). In addition, univariate analysis of systemic cotreatments revealed no significant association with elevated RP risk (Fig. 2). No significant added risk for RP from single-agent endocrine treatment (aromatase inhibitors, tamoxifen, goserelin) was detected. Within the entire patient cohort, the combination of tamoxifen and GnRH agonists significantly raised the risk of RP (OR 2.61; 95% CI 1.31–5.22). Controlling age as a confounding factor, the actual risk of developing any-grade RP upon combined tamoxifen and goserelin cotreatment was OR 4.38 (95% CI 2.06–9.32). No interference between this combination and RP risk was detected by including smoking status as a second potentially confounding factor.

**Fig. 3:**
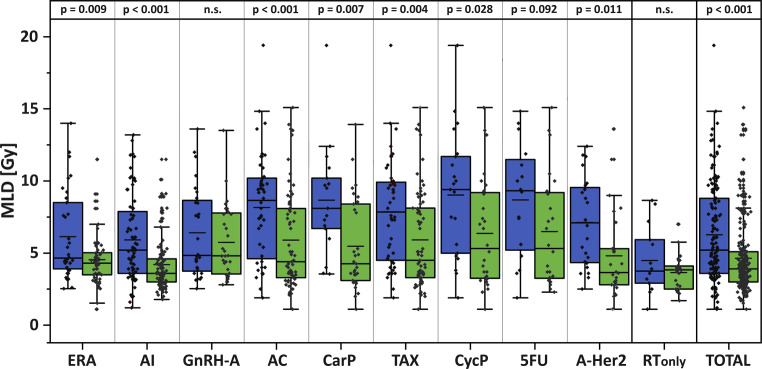
Comparison of RP development depending on systemic therapies and MLD of radiation treatment. *Blue boxplots* summarize the MLD data points of patients with any-grade RP after treatment. *Green boxplots* represent the MLD distribution of patients not afflicted by any-grade RP. Boxes include the 25th to 75th percentiles, whisker = 1.5 interquartile ranges, median (*full line*), and mean (*short line*) MLD. Dataset is depicted for nine different classes of substances used for systemic treatment. In addition to RT, 211 patients received a single systemic agent as an adjuvant, a neoadjuvant, or a concomitant approach. 137 patients were treated by combined systemic schedules including different classes of substances and were separately analyzed for each class of chemoradiation. *ERA* estrogen receptor antagonists, N_total/ag-RP_ = 83/34, *AI* aromatase inhibitors, N_total/ag-RP_ = 185/76, *GnrH‑A* gonadotropin-releasing hormone antagonist, N_total/ag-RP_ = 60/28, *AC* anthracycline, N_total/ag-RP_ = 109/46, *CarP* carboplatin, N_total/ag-RP_ = 48/17, *TAX* taxane, N_total/ag-RP_ = 117/48, *CycP* cyclophosphamide, N_total/ag-RP_ = 49/21, *5FU* 5-FU, N_total/ag-RP_ = 40/16, *A‑Her2* anti-Her2 antibody, N_total/ag-RP_ = 50/24, *RT*_*only*_ radiation monotherapy, N_total/ag-RP_ = 41/14, *TOTAL* all RP/ARP and non-afflicted patients, N_total/ag-RP_ = 396/151

The role of mean lung dose in independently promoting any-grade RP is summarized in Fig. 3. Regardless of systemic treatment variations, MLD is significantly higher (rise of mean MLD = + 35.3%; *p* < 0.001) in the cohort of patients developing symptomatic (*N* = 40) or any-grade RP (*N* = 151) than in the cohort without RP (*N* = 245). Separate analysis of patient cohorts receiving different combined systemic treatments revealed that MLD was significantly higher in nearly all sub-cohorts of patients developing any-grade RP: estrogen receptor antagonists (+35.3%; *p* = 0.009), aromatase inhibitors (+39.8%; *p* < 0.001), anthracyclines (+40.4%; *p* < 0.001), carboplatin (+53.8%; *p* = 0.007), taxanes (+34.6%; *p* = 0.004), cyclophosphamide (+43.3%; *p* = 0.028), 5‑FU (+33.8%; *p* = 0.092), and/or with anti-Her2 antibodies (+56.7%; *p* = 0.011). For patients treated with a gonadotropin-releasing hormone antagonist, only a minimally elevated mean MLD (+11.5%, not significant) was recorded in the sub-cohort afflicted by any-grade RP. In the small subgroup of RP patients treated with radiation monotherapy only, a higher mean MLD (+23.0%) was found, albeit statistically not significant.

## Discussion

Acute radiation pneumonitis and radiation recall pneumonitis typically arise from irradiated tumors of the lung but may incur after irradiation of other tumors located anywhere in the thoracic region, particularly following breast cancer treatment [27]. Treatment-associated pneumonitis can also be induced by a great variety of antineoplastic drugs. Among such chemical triggers, classical chemotherapeutics as well as novel anticancer agents have been identified [3]. However, in case of combined cancer treatment schedules, the specific interaction of systemic agents and radiotherapy as well as the quantitative contribution of each to trigger pneumonitis is still a matter of investigation. In the past, often-contradicting studies are characterized mainly by their retrospective nature, missing systematic analysis of substance classes, primary focus on one therapeutic only, and by smaller patient cohorts.

Generally, patients who receive combinations of certain cytotoxic agents are thought to exhibit a higher frequency of lung toxicity [3]. Potentially harmful adjuvant drug combinations might additionally increase the risk of radiation-induced pneumonitis.

In the present study, we prospectively enrolled 396 breast cancer patients to investigate the distinct impact of various systemic therapeutics and of treatment-associated co-irradiation of lung tissue on the risk of RP development. The study included patients who received adjuvant radiation therapy of the breast and chest-wall region—alone or including locoregional nodal areas—thus being a representative cohort of routine breast RT. There were no restrictions for enrolment regarding the indicated schedules of adjuvant systemic treatment, thus including different classes of endocrine, chemotherapeutic, and targeted agents, as well as of antibodies. The study also included a subgroup of patients receiving radiation therapy only. We performed systematic monitoring for lung toxicity using CT scans and blood analysis after 3 and 6 months for all patients. This setup allowed us to discriminate the proportional risk contribution of individual classes of therapeutics as well as the impact of radiotherapy itself on the development of treatment-associated pneumonitis (symptomatic and asymptomatic). We did not, however, include pulmonary fibrosis occurring 24 months or later after RT as an additional study endpoint.

Remarkably, neither chemotherapeutics, endocrine therapies, targeted agents, and antibodies, nor most of their combinations (see Table 1) significantly increased the risk of inducing pneumonitis in our breast cancer patients. In fact, overall radiation exposure of lung tissue in the applied sequential treatment schedules, expressed as mean lung dose (MLD), proved the most prominent and most significant factor in determining RP risk, thereby confirming previous findings [11, 28]. Our study further confirms age and smoking status as patient-specific and commonly accepted factors significantly influencing RP risk.

Among the chemotherapeutics most associated with inducing RP, especially taxanes have been identified, either alone [29] or as part of combined schedules with other agents, as reported by several studies and case reports in the past [18–20, 29–31]. However, our recent and previous findings [11] regarding the absence of clinically relevant RP induced by taxanes are supported by other studies like that of Yu et al. [20], who investigated a cohort of 524 breast cancer patients receiving FAC (5-FU, doxorubicin, cyclophosphamide) with or without paclitaxel and subsequent RT. The lacking evidence for taxanes further promoting RP significantly, as also demonstrated in our present study of prospective nature, might be attributed to varying rationales of applied chemoradiation. In fact, the available literature mainly reports on a more deleterious effect on lung tissue of concomitant schedules rather than in the sequential taxane–RT treatment of breast cancer. Consequently, a radiosensitizing effect of taxanes to the detriment of lung tissue as earlier reported for concomitant chemoradiation [18] is not apparent in the sequential protocols applied in this study. In addition, the distinct influence of lung tissue co-irradiation could not be precisely described by the surrogate parameter CLD during early toxicity studies. Nowadays it is commonly accepted that volumetric dosimetry (MLD, V_20Gy_, V_30Gy_) is much more accurate in predicting lung toxicity of RT, thus finally allowing differentiation between the distinct contribution of systemic therapy and of RT, as presented herein. In conclusion, no significant additive effect of taxanes and co-irradiation of lung tissue (as part of sequential adjuvant schedules) was detected in our cohort of 396 breast cancer patients. Thus, MLD turned out to be the only significant treatment-related predictive factor of any-grade radiation pneumonitis in the group of taxane-cotreated patients.

Analogous findings and conclusions can be drawn from our data regarding the role of the other chemotherapeutics (cyclophosphamide, carboplatin, 5‑FU, doxorubicin, and epirubicin) in their potential to trigger RP in our exclusively sequential chemoradiation schedules. The pulmonary toxicity associated with each of these drugs alone is known to be moderate, even if administered in combination schedules or prior to subsequent RT [27, 32, 33]. A potential radiosensitizing effect of these drugs on the co-irradiated lung tissue could also not be identified for any of the combined schedules upon our sequential adjuvant RT regimen. Even in the combination of radiosensitizing 5‑FU with taxanes [3], no elevated risk of lung toxicity was observed, and none of these 36 patients developed symptomatic RP.

Beside classical chemoradiation, additional cotreatment with endocrine and antibody therapeutics is standard of care in breast cancer treatment of eligible patients. In accordance with the absence of reported evidence in the literature [34], we could not detect a significant added risk for developing RP from aromatase inhibitor treatment (OR 1.20, 95% CI 0.80–1.81, n. s.). Estrogen receptor antagonists are also rarely associated with early pneumonitis induction, although tamoxifen is considered a possible promoter of later occurring pulmonary fibrosis as well as of acute pneumonitis [22, 35, 36]. Our prospective data do not evidence a significant overall risk enhancement for pneumonitis following adjuvant tamoxifen treatment (OR 1.16, 95% CI 0.71–1.90, n. s.) if compared to all other treatment subgroups in our patient cohort. Varga et al. [36] reported a more elevated but also not significantly increased risk for any-grade RP (OR 1.68) in their prospective cohort of 328 breast cancer patients cotreated with tamoxifen in comparison to RT alone. Further investigation including even larger patient cohorts could finally give conclusive evidence of the role of tamoxifen alone in RP induction.

Regarding GnRH analogues, uncommon interstitial lung disease is a known side effect of goserelin with a reported frequency of < 1%. In the present study, GnRH analogues alone exhibited a moderately increased potential in pneumonitis promotion after breast cancer RT, albeit not to a significant level (OR 1.52, 95% CI 0.87–2.67). However, among all investigated systemic treatments, the risk of RP in the goserelin subgroups was least dependent on MLD. As reported by us previously from a much smaller cohort of 100 patients [11], occurrence of symptomatic RP appeared to be associated with concomitant treatment of goserelin and tamoxifen. In this larger prospective evaluation including 396 patients, the combination of tamoxifen and GnRH agonists proved to markedly and significantly raise the risk of RP (OR 2.61, 95% CI 1.3–5.2). The incidence of any-grade RP in patients receiving both tamoxifen and goserelin concomitantly to RT thereby increases from 35.9 to 59.5%, and of symptomatic RP from 8.9 to 21.6%, if compared to all other treatment schedules not including this combination of drugs. Since this combination of drugs is predominantly used in premenopausal patients, age has to be considered in the evaluation of the actual risk probability for this subgroup of patients in comparison to all others in the cohort (mean age 46.0 vs. 58.5 years). When accounting for age as a confounding factor, the actual risk of developing an any-grade RP even escalates to an OR of 4.38 (95% CI 2.06–9.32). To our knowledge, the combined role of younger age, tamoxifen, and goserelin in promoting RP risk has never been systematically analyzed and may warrant further investigation. Moreover, and to our knowledge, the biomolecular mechanism(s) involved in lung tissue complications upon administration of RT combined with LHRH analogs and estrogen receptor antagonists is not understood and needs specific research. However, our clinical findings already indicate that special attention should be paid to monitoring for signs of lung toxicity after RT in these patients.

There is evidence that also monoclonal antibody HER‑2 receptor antagonists (trastuzumab, pertuzumab) can increase the risk of pneumonitis autonomously [37]. In fact, these antibodies demonstrated a moderately increased potential in pneumonitis induction after breast cancer RT also within our cohort of patients, but not in a significant manner (OR 1.48, 95% CI 0.83–2.63). Except for the MLD of co-irradiation, no other synergistic relation with combined systemic treatments could be identified.

Finally, the sub-cohort receiving RT alone (without any systemic adjuvant treatment) mainly comprises patients with early-stage breast cancer, thus receiving RT of the breast and chest wall only. This treatment is normally characterized by significantly lower volumes in lung exposure to radiation, which is expressed by mean MLD values of ≤ 5 Gy. Because of minimal MLD variances within the entire sub-cohort of patients treated with RT alone, no significantly elevated MLD could be evidenced in patients who exhibited any-grade RP. As expected, the risk of RP increased significantly when lymph node irradiation was indicated, expressed as symptomatic RP incidences increased by a factor of 1.6 (PCN inclusion) and 2.6 (PCN + IMN inclusion). Due to the applied moderately higher dose concepts used during the study, the observed overall RP incidence was higher than reported for comparable patient cohorts, especially as IMRT/VMAT is commonly performed nowadays [38–42]. However, in our cohort, all 40 patients with diagnosed RP ≤ grade II exhibited moderate symptoms, and all of them recovered completely within a median time of 9 months (range 1 to 24 months).

## Conclusion

Several patient-specific and treatment-related factors are suspected or known to increase the RP risk after breast-conserving surgery of breast cancer followed by RT. Particularly, the role of various combined adjuvant endocrine, antibody, and sequential chemotherapeutic treatments in triggering RP is often unclear, and therefore demands specific investigations. The systematic analysis performed in this prospective study reveals that none of the chemotherapeutic agents administered before RT, including taxanes, significantly elevate the RP risk, even when using combined protocols. Furthermore, mean lung dose is the single most important determinant of RP. While no endocrine treatment alone exhibited a significant additional risk, the combination of goserelin and tamoxifen proved similarly impactful to higher MLD. Thus, we highly recommend close monitoring of the respective breast cancer patients in order to ensure timely diagnosis and treatment of early lung toxicity, thereby preventing progressive organ impairment, such as pulmonary fibrosis or organizing pneumonia.
